# Long-term outcomes of pericardiectomy for constrictive pericarditis

**DOI:** 10.1186/s13019-015-0385-8

**Published:** 2015-11-27

**Authors:** Murat Biçer, Bülent Özdemir, İris Kan, Ahmet Yüksel, Mustafa Tok, Işık Şenkaya

**Affiliations:** 1 Department of Cardiovascular Surgery, Uludağ University Medical Faculty, Bursa, Turkey; 2 Department of Cardiology, Uludağ University Medical Faculty, Görükle Kampüsü, Nilüfer, Bursa, 16000 Turkey

**Keywords:** Constrictive pericarditis, Pericardiectomy, postoperative results

## Abstract

**Background:**

Constrictive pericarditis is a rare and disabling disease that can result in chronic fibrous thickening of the pericardium. The purpose of this study was to evaluate the long-term outcomes following treatment of constrictive pericarditis by pericardiectomy.

**Methods:**

Between September 1992 and May 2014, 47 patients who underwent pericardiectomy for constrictive pericarditis were retrospectively examined. Demographic, pre-, intra- and postoperative data and long-term outcomes were analyzed.

**Results:**

Thirty of the patients were male, the mean age was 45.8 ± 16.7. Aetiology of constrictive pericarditis was tuberculosis in 22 (46.8 %) patients, idiopathic in 15 (31.9 %), malignancy in 3 (6.4 %), prior cardiac surgery in 2 (4.3 %), non-tuberculosis bacterial infections in 2 (4.3 %), radiotherapy in 1 (2.1 %), uraemia in 1 (2.1 %) and post-traumatic in 1 (2.1 %). The surgical approach was achieved via a median sternotomy in all patients except only 1 patient. The mean operative time was 156.4 ± 45.7 min. Improvement in functional status in 80 % of patients’ at least one New York Heart Association (NYHA) functional class was observed. In-hospital mortality rate was 2.1 % (1 of 47 patients). The cause of death was pneumonia leading to progressive respiratory failure. The late mortality rate was 23.4 % (11 of 47 patients). The mean follow-up time was 61.2 ± 66 months. The actuarial survival rates were 91 %, 85 % and 81 % at 1, 5 and 10 years, respectively. Recurrence requiring a repeat pericardiectomy was developed in no patient during follow-up.

**Conclusion:**

Pericardiectomy is associated with high morbidity and mortality rates. Cases with neoplastic diseases, diminished cardiac output, cases in need of reoperation are expected to have high mortality rates and less chance of functional recovery.

## Background

Constrictive pericarditis is caused by thickening of the pericardium. It causes diastolic dysfunction and at the end the filling of heart is impeded by the constricted pericardium surrounding the heart. The aetiology is idiopathic, prior cardiac surgery, postradiotherapy , postinfective, connective tissue disease-related, neoplastic, uremic, sarcoidosis, and miscellaneous [1, 2]. After occurrence of constriction; the symptoms related to fluid overload and reduced cardiac output are progressive in nature.

The study conducted by Ling et al. revealed that the majority of patients presented with congestive heart failure. With decreasing frequencies the patients had presented with chest pain, abdominal symptoms, cardiac tamponade, atrial arrhythmia and frank liver disease [3].

Definitive treatment for chronic constrictive pericarditis is pericardiectomy. In this paper we retrospectively examined the data of pericadiectomies of 47 cases that were operated between 1992 and 2014.

## Methods

We evaluated the data regarding patients that had pericardiectomy due to having constrictive pericarditis in the Research Hospital of Uludağ University Medical Faculty between September 1992 and May 2014. Demographic and operative data were evaluated. The outcomes of the patients were noted.

All patients underwent pericardiectomy via median sternotomy except for one case. A left anterior thoracotomy was performed in that case. In patients approached via sternotomy total pericardiectomy was performed between the two phrenic nerves and from the great vessels to the basal aspect of the heart. Partial pericardiectomy was achieved in one case that underwent left anterior thoracotomy.

One patient had concomitant mitral valve replacement and 4 patients required concomitant coronary bypass surgery. Cardiopulmonary bypass (CPB) was not used during coronary artery bypass grafting except for 2 patients (off-pump technique). One patient was started with CPB and one patient was later converted to CPB due to cardiac injury. All patients were monitored with Swan-Ganz catheterization.

Postoperative death was defined as death occurring within 30 days of operation or within the hospitalization period for the operation.

Continuous variables were expressed as the mean with standard deviation and categorical variables as percentages. The chi-squared test and the Student’s t-test were performed as appropriate. Survival was assessed by the Kaplan-Meier method. The Wilcoxon’s signed rank test was used to compare the NYHA functional classes of patients preoperatively and postoperatively. A *p* value < 0.05 was considered statistically significant. Statistical analysis was performed using IBM SPSS Statistics Version 20.

## Results

Forty-seven patients had operation for constrictive pericarditis. Mean age was 45.8 ± 16.7 for the patients and 30 were males. The baseline characteristics of the patients are given in Table 1. Dyspnoea was the most common complaint of the patients. Peripheral oedema was also most commonly noted during physical examination. The majority of the cases had NYHA functional class II and III. The functional class was improved at least one NYHA functional class in 80 % of the cases. The most common etiologic factor for our patients was tuberculosis. Idiopathic constrictive pericarditis ranked second (Table 2). In-hospital mortality rate was 2.1 % (1 of 47 patients). The cause of death was pneumonia secondary to progressive respiratory failure. The late mortality rate was 23.4 % (11 of 47 patients). The mean follow-up time was 61.2 ± 66 months. The longest survival was 214 months. The actuarial survival rates were 91 %, 85 % and 81 % at 1, 5 and 10 years, respectively.

**Table 1:** Preoperative characteristics of the patients

Variable	Result
Mean age (years)	45.8 ± 16.7
Male/Female ratio	30/17
Symptoms (no. of patients, %)	
Dyspnoea	34 (72.3 %)
Lower limb oedema	15 (31.9 %)
Chest pain	10 (21.3 %)
Abdominal distension	7 (14.9 %)
Palpitation	3 (6.4 %)
Constitutional (Fever, fatigue, weight loss, etc.)	19 (40.4 %)
Signs (no. of patients, %)	
Peripheral oedema	20 (42.5 %)
Jugular venous distension	16 (34.0 %)
Hepatosplenomegaly	11 (23.4 %)
Ascites	9 (19.1 %)
Pericardial knock	6 (12.8 %)
Pulsus paradoxus	3 (6.4 %)

**Table 2:** Aetiologies of constrictive pericarditis

Etiological factor	No. of patients (%)
Tuberculosis	22 (46.8 %)
Idiopathic	15 (31.9 %)
Malignancy	3 (6.4 %)
History of CABG operation	2 (4.3 %)
Non-tuberculosis bacterial infections	2 (4.3 %)
Radiotherapy	1 (2.1 %)
Uraemia	1 (2.1 %)
Post-traumatic	1 (2.1 %)

CABG: Coronary artery bypass grafting

Mean duration of the pericardiectomy operation was 156.4 ± 45.7. Cardiopulmonary bypass utilization was not high (12.8 %). The mean preoperative central venous pressures of the cases decreased significantly from 17.0 ± 2.9 to 10.8 ± 2.1 postoperatively (The data regarding operation, post operative care and treatment are given in Table 3). The postoperative complications noted were pleural effusion, pulmonary infection, long-term intubation, low output syndrome, bleeding (requiring surgical revision), acute renal failure, hepatic failure, and wound infection. The frequencies are given in Table 4.

**Table 3:** Intraoperative and postoperative data of the patients

Variable	Result
Mean operation time (minutes)	156.4 ± 45.7
CPB utilization (no. of patients, %)	6 (12.8 %)
Concomitant procedures (no. of patients, %)	
CABG	4 (8.6 %)
MVR	1 (2.1 %)
CVP change	
Preoperative CVP (mmHg)	17.0 ± 2.9
Postoperative CVP (mmHg)	10.8 ± 2.1
Inotropic support (no. of patients, %)	11 (23.4 %)
Low dose	8 (17.0 %)
Medium-high dose	3 (6.4 %)
Blood product requirement	
No. of patients, %	26 (55.3 %)
Mean amount used (units)	1.9 ± 1.2
Mean duration of mechanical ventilation (hours)	28.5 ± 80.1
Mean length of ICU stay (days)	2.8 ± 4.6
Mean length of hospital stay (days)	9.2 ± 7.7
In-hospital mortality (no. of patient, %)	1 (2.1 %)

CPB: Cardiopulmonary bypass, CABG: Coronary artery bypass grafting, CVP: Central venous pressure, MVR: Mitral valve replacement, ICU: Intensive care unit

**Table 4:** Early postoperative complications

Complication	No. of patients (%)
Pleural effusion	6 (12.8 %)
Pulmonary infection	4 (8.5 %)
Long-term intubation (over 48 hours)	4 (8.5 %)
Low output syndrome	3 (6.4 %)
Bleeding (requiring surgical revision)	1 (2.1 %)
Acute renal failure	1 (2.1 %)
Hepatic failure	1 (2.1 %)
Wound infection	1 (2.1 %)

## Discussion

**Fig. 1:**
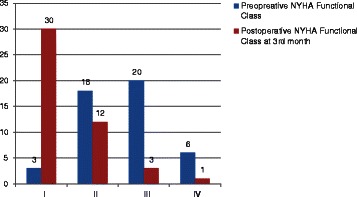
Functional Status of patients pre- and postoperatively

The preoperative functional class of the patients in majority belonged to NYHA Class II and III like previously reported by Ghavidel et al. [4]. The comparison of pre and post operative NYHA functional class of all the patients is given in Fig. 1. Dyspnoea and oedema was the most frequent symptoms. The constriction affects both ventricles and causes both left and right ventricular dysfunction but symptoms of right heart failure dominate. The congestive heart failure in the presence of normal left ventricular systolic functions should make us to think about constrictive or restrictive type pathologies. Also discrimination between restrictive cardiomyopathies and constrictive pericarditis is important. History, physical examination, electrocardiography, chest radiography, echocardiography, cardiac CT or MRI, and hemodynamic evaluation all are important modalities used for the diagnosis [5]. Physical examination revealed peripheral oedema, jugular venous distension, hepatosplenomegaly and ascites in decreasing frequency in our study. Physical examination findings commonly reported in the literature involve elevated jugular venous pressure (being most common), oedema, ascites, pulsus paradoxus, Kussmaul's sign, and pericardial knock [1, 3].

In this study, our patients had tuberculosis and idiopathic causes as etiological factors. Also encountered causes were malignancy, prior cardiac operation, and bacterial infection other than tuberculosis, radiotherapy, tuberculosis and trauma. In another report by Avgerinos; idiopathic, postoperative, post-radiation, and tuberculosis were found in decreasing frequency [6]. Talreja et al. in their study that included 143 patients with proven constriction who underwent pericardiectomy between 1993 and 1999, and compared the patients according to the thickness of the pericardium. In their study both the patients with normal pericardial thickness (≤2 mm) and patients with thickened pericardium (>2 mm) had frequently the aetiology of a previous cardiac operation [7]. But interestingly; in this study idiopathic disease was the most frequent disease in the group with thickened pericardium.

In our study the mean central venous pressure decreased from 17.0 ± 2.9 to 10.8 ± 2.1. In a study included patients that had pericardiectomy due to constriction the central venous pressure significantly decreased from 15.3 ± 3.7 mmHg to 8.8 ± 3.1 [8]. Postoperative mortality occurred in one patient as in-hospital mortality due to pneumonia. Late mortality was 17.4 %. Bertog et al. reported a perioperative mortality of 6 % in their study. In his study idiopathic constrictive pericarditis had the best prognosis [2]. Lin et al. reported an in-hospital mortality of 3.9 %. In his study; low cardiac output syndrome due to right heart failure and acute renal failure were the causes of mortalities [9]. In 47 pericardiectomy patients a 30-day mortality of 8.5 % occurred and 69 % of cases clinical improved with echocardiographic parameters normalizing eventually [10].

**Fig. 2:**
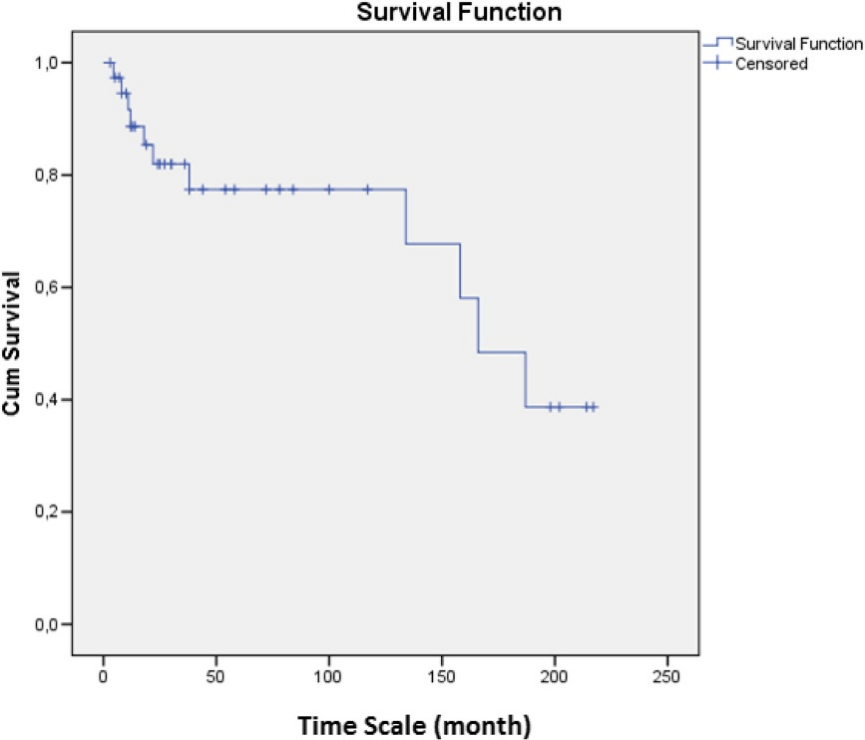
Cumulative Survival of the patients

In our series all the cases with malignancies died due to the primary disease. Idiopathic group (n = 11) has no fatalities with the maximum follow up of 84 months in one patient. The patients with tuberculosis (n = 17) has a mortality rate of 23,5 %. Late mortality rates of patients with tuberculosis that had pericardiectomy were at 5 and 10 years were 1.6 % and 9.7 %, respectively in a study conducted by Çınar et al. [11]. The cumulative survival data are given in Fig. 2. Long term survival of pericardiectomy operations is dependent on the aetiology of the constrictive pericarditis. High mortality rates in cases with malignancies and patients that had radiotherapy are expected. In a small series of patients a mean survival of 14.82 ± 4.4 months was reported in pericardiectomy patients that had constrictive pericarditis secondary to neoplastic disease [12] The survival of pericardiectomy is also related to completeness of the resection at first, otherwise a second operation with a high mortality risk may be obligatory [6].

## Conclusion

Pericardiectomy is associated with high morbidity and mortality rates. The variability of the mortality rates is due to study groups belonging to different aetiological diseases. Cases with neoplastic diseases, diminished cardiac output, cases in need of reoperation are expected to have high mortality rates and less chance of functional recovery.
